# Agronomical Practices and Management for Commercial Cultivation of *Portulaca oleracea* as a Crop: A Review

**DOI:** 10.3390/plants12061246

**Published:** 2023-03-09

**Authors:** Angel Carrascosa, Jose Antonio Pascual, Margarita Ros, Spyridon A. Petropoulos, Maria del Mar Alguacil

**Affiliations:** 1 CSIC-Centro de Edafología y Biología Aplicada del Segura, Department of Soil and Water Conservation, Campus de Espinardo, P.O. Box 164, 30100 Murcia, Spain; 2 Department of Agriculture, Crop Production and Rural Environment, University of Thessaly, Fytokou Street, 38446 Volos, Greece

**Keywords:** purslane, wild edible species, stress tolerance, nitrogen application, organic amendment, farming systems

## Abstract

**Featured Application:**

***Portulaca oleracea* is a valuable wild species, spread worldwide, and is consumed in different regions of the world. As a wild edible species, it is a promising alternative to substitute conventional crops in harsh conditions due to its resilience to adverse conditions. In the present review, we tried to compile the most up-to-date literature regarding the management and cultivation practices of *Portulaca oleracea.* The presented information will be useful to farmers and stakeholders for the commercial cultivation of the species and its introduction as a complementary/alternative crop in the current production systems, while at the same time could be used for the phytoremediation and reclamation of degraded and abandoned agricultural land.**

**Abstract:**

Soil is an essential resource, and its degradation is challenging modern agriculture, while its impact is expected to increase in the near future. One of the strategies to address this issue is to incorporate new alternative crops able to tolerate arduous conditions, as well as for the use of sustainable agricultural practices in order to recover and/or improve soil health. Additionally, the increasing market for new functional/healthy natural foods promotes the search for potential alternative crop species with promising bioactive compounds content. For this purpose, wild edible plants are a key option because they have already been consumed for hundreds of years in traditional gastronomy and there is well-established evidence of their health-promoting effects. Moreover, since they are not a cultivated species, they are able to grow under natural conditions without human intervention. Among them, common purslane is an interesting wild edible species and a good candidate for integration in commercial farming systems. With worldwide spread, it is able to tolerate drought, salinity and heat stress and is already used in traditional dishes, while it is highly appreciated for its high nutritional value due to its bioactive compound content, especially omega-3 fatty acids. In this review, we aim to present the breeding and cultivation practices of purslane, as well as the effects of abiotic stressors on yield and chemical composition of the edible parts. Finally, we present information that helps to optimize purslane cultivation and facilitate its management in degraded soils for their exploitation in the existing farming systems.

## 1. Introduction

The world population is estimated to reach 10 billion habitants by the year 2050. As the population increases and more people currently with problems concerning food security will have access to better nutrition, the demand for food production will significantly increase [1,2]. On the other hand, modern agriculture has depended on intensive agriculture practices during the last decades aiming to achieve the high yields required to fulfil the current food demand; however, the increase on agricultural productivity necessary to supply the needed food demand might not improve enough in the future [3,4].

There are different environmental factors that are detrimental to agriculture, such as the increase of temperatures or the reduction of precipitations [1,2,3,4,5], along with different types of degradation of soils (salinization, nutrient depletion, soil organic carbon depletion or reduced water holding capacity) [5]. Thirty-three percent (33%) of land destined for agriculture globally is affected to some extent by degradation [6], whereas 25% is already considered degraded [6,7]. Some important agricultural regions in the world are even more sensitive to aridization, such as the Mediterranean basin, West Asia or North America [7,8].

In order to revalorize and reclaim degraded agricultural soils, the introduction of new cultivars/crops that are well-adapted to natural conditions and easy to manage has been proposed [9]. This is the case of wild edible plants (WEPs), which are native or naturalized plant species able to grow in natural conditions without human intervention and traditionally used as a food source or as complementary ingredients in local recipes, or even as ’famine foods’ [10,11,12]. The availability of WEPs depends on the area and the growing conditions, while the cultivation of WEPs adapted to scarcity of water, high salinity and high temperatures in the summer are the most interesting features for the Mediterranean basin conditions [13]. The successful integration of WEPs in cropping systems, thanks to their easy and low-input cultivation, might allow farmers not only to diversify their crops in harsh environmental conditions but also, along with the implementation of sustainable agronomic practices, to recover and improve the soil quality [10,14].

Apart from the agronomic aspect, the nutritional value of WEPS also has to be considered. During the last decades, changes in human diet and health care, such as overconsumption of calories or meat and reduced physical activity, have increased the incidence of chronic diseases [11,15]. This has increased the interest of society in healthy diets, resulting in new market trends for healthy foods with high nutritional and organoleptic properties, such as the ones that can be offered by WEPs [13,16]. Therefore, the current drivers may provide novel opportunities for both small and large food industries to produce new heathy food products, while small-scale farms and rural communities may also benefit from these trends [17,18].

*Portulaca oleracea* (L.) or common purslane is an annual prostate or erect species, with succulent, branched and reddish stems [19]. It is a herbaceous succulent annual plant member of the Portulacaceae family [20]. Purslane can complete its life cycle in 2–4 months and has the ability to re-root after hoeing when stems remain moist [9]. Purslane is also known by its tolerance to stressors, since it is considered a halophyte plant able to endure in moderate salinity conditions [21,22]. Moreover, it has a C4 metabolism and is able to switch to a CAM-like metabolism under stress, a feature that improves its water use efficiency, making purslane a highly competitive alternative in arid lands, with scarcity of water and high temperature conditions [23,24,25]. It is considered a wild edible plant distributed worldwide, and one of the three most frequently reported weeds across the world. It can be easily grown in warm and dry places, and it is widely distributed in the Mediterranean basin, Asia, the Caribbean, North America, México and Australia. Although it is considered a noxious weed that affects conventional crops in many regions of the world, it is also traditionally consumed in Spain, Greece, Italy, Turkey, the USA and China among others [20,26]. The appreciated parts of purslane are fresh leaves and stems, with a distinctive sour taste [14,27,28]. They are mainly used in fresh green salads, but they also can be pickled or cooked [29]. However, the special attention that purslane has received during recent years is due to its exceptional nutritional content, mainly because of its high content of omega-3 fatty acids; antioxidant compounds such as vitamin A, C, E and B; and minerals such as potassium, calcium and magnesium [30], especially when plants are grown under stress conditions where higher concentrations of beneficial compounds are found [31]. These compounds are associated with the pharmacological properties of the species, such as its antioxidant, anti-inflammatory, antidiabetic, anti-obesity and hepatoprotective potential among others [32], which have led some to consider purslane a ´food of the future´ [13,33,34].

As a wild plant, it is considered an easy to grow crop under field conditions in warm places due to its natural tolerance to drought and heat. Additionally, the nutritional needs of purslane are considered low, since low inputs of nitrogen in soil (~60 kg ha^−1^) can lead to high responses in yield [35,36]. Depending on edaphoclimatic conditions, fertilization rates, plantation density and harvesting time, producers may expect variable yields which may range between 13 and 100 ton ha^−1^ [37,38,39]. The production of purslane in hydroponic and floating systems with different substrates and fertilization rates has also been studied, which allows high plantation densities, shorter growing periods and expected fresh yields between 2 and 6 Kg m^−2^ [36,40,41]. For these reasons, the interest in purslane cultivation, besides its nutritional properties, is due to its capacity to thrive in harsh edaphoclimatic conditions where conventional crops are striving to grow properly [24,42,43]. Therefore, the concerns about the long-term sustainability of farming systems are increasing, especially the management and conservation of soil quality. Sustainable agricultural practices have been reported to increase soil organic matter and water-holding capacity. They also improve soil structure and increase permeability, while they decrease N leaching compared to soils managed exclusively by conventional farming regimes [44]. Organic matter plays a key role in soil quality and soil ecosystems since it provides the necessary substrate for decomposing microbes, thus increasing the activity of soil microbes that supply mineral nutrients to plants, enhancing plants’ tolerance to stresses and consequently increasing plant growth and yield [45,46,47]. However, the benefits from the transition to sustainable and low-input farming management may take several years to be detectable in terms of increased SOM quantity and quality, which are associated with soil fertility in the long-term [48]. In this scenario, the integration of wild edible plants such as purslane in the existing farming systems may contribute directly to the improvement of soil organic carbon through practices such as mulching or indirectly via enriching soil microbiomes involved in organic matter decomposition [49,50]. Moreover, the reclamation of degraded soils should be achieved through phytoremediation processes, since purslane has been proved to be able to remove contaminants from soils and gradually contribute to the recovery of such soils [51,52,53].

Soil-improving practices such as the addition of organic amendments like animal and green manure or guano have traditionally been used in agriculture for a long time. These were the main methods to improve soil until the appearance and establishment of chemical fertilizers in the Green Revolution in the 1950s. Today, the most common organic amendments can be classified into five categories: animal manure, municipal biosolids and sewage; green manure and crop residues; food residue and waste; waste from manufacturing processes; and compost [54]. Although the transition to organic agriculture and the establishment of sustainable agricultural practices may enhance agricultural systems in the long-term conditions, in already degraded soils where conventional crops are hard if not possible to be cultivated, organic agriculture may not be enough to recover soil quality while maintaining economic viability for farmers at the same time [55]. Therefore, it may be interesting to implement new highly tolerant, easy to grow crops such as purslane to help farmers recover degraded soils, as they can obtain profitability.

The objectives of this review were to conduct a bibliometric analysis for the different agronomical practices and management of purslane in order to define the conditions that are most suitable to enhance purslane yield and integrate the species in both conventional and sustainable cropping systems.

## 2. Materials and Methods

Data collected from Scopus, PubAg, Agris, PubMed and Science Direct databases for the 2007–2022 period were reviewed and analyzed using smart tools and Boolean (AND, OR and NOT) and proximity operators. The central axis descriptor was ‘*Portulaca oleracea*’ OR ‘purslane’ using the search field ‘Article title’. Key- or co-words related to the cultivation of purslane were searched using the search file ‘Article title, Abstract, and Keywords’ resulting in a total of 135 articles. Key- or co-words searched were: ‘Fertilization’ (12 articles), ‘Nitrogen fertilization’ (8 articles), ‘Germination’ (50 articles), ‘Manure’ (5 articles), ‘Arbuscular Mycorrhiza’ (2 articles), ‘Harvesting AND Stage’ (11 articles), ‘Intercropping’ (1 article), ‘Drought’ (38 articles), ‘salinity’ (65 articles) and ‘heat’ (28 articles). We selected the articles that studied purslane growth and yield, as well as the propagation and nutritional properties of the species. The results presented are only those referring to common purslane.

## 3. Cultivation Practices

Although purslane is considered a noxious weed due to its relatively easy propagation, it is important to consider the best cultivation practices that are necessary to define its propagation and cultivation requirements. Moreover, this information will be useful for the farmers in order to commercially cultivate the species and produce final products of quality in terms of visual appearance, chemical composition and bioactive properties. The following sections compile the data available in the literature regarding the most common cultivation practices of purslane.

### 3.1. Propagation and Growing Conditions

Purslane is an annual plant that usually grows during the hottest months of the year and can complete its life cycle (from sowing to seed) in approximately 2–4 months [56]. The flowers bloom from June to September for a few hours in the morning. The seeds are formed in small capsules which contain many brownish-to-black seeds up to 0.5 mm in diameter [19]. Seeds germinate very easily, and under suitable conditions, the germination rate can go up to 90% in 24 h after watering [57]. Purslane is very resilient to adverse conditions and 82.4% of seeds are able to germinate at a 20 cm depth after one year [26]. Increasing temperatures up to 25 °C are associated with high germination rates, whereas excessive temperatures of 50 °C may result in thermal death [26,58]. Moreover, the species shows a high variability of seed dormancy which allows the avoidance of cold temperatures while maintaining its vigor. However, although Feng et al. [26] suggested that purslane seeds collected from China showed dormancy, other studies did not report any dormancy effects [20,59]. Moreover, Feng et al. [26], who tested the effect of storage on seed germination rate, reported that storage at −20 °C for one year, as well as storage at 45 °C for 60 days, resulted in the highest germination percentage. In contrast, Chauhan and Johnson [56] reported that seed germination of purslane is not affected by the storage duration but is strongly stimulated by light. In their experiment, they evaluated germination rates under dark and light/dark conditions (12/12 h) and different temperature regimes, and they found that only a small proportion of seeds were able to germinate in darkness, despite the temperatures, whereas under the light/dark cycle, the germination rates varied, reaching 70% (25/15 °C), 75% (35/25 °C) and 81% (30/20 °C). Moreover, the same authors did not find any differences in the germination rates of seeds stored for 0 to 6 months at 30/20 °C (day/night), regardless of the lighting regime (light/dark or dark) [56]. Another factor that affects the seed germination rate is seed burial depth, with germination rates showing an exponential decrease with increasing depth (0% germination rate at depths higher than 2 cm), although Benvenuti et al. [60] recorded rates of 12.2% at depths of 6 cm. The tillage system may also have an impact on seed germination with minimum tillage significantly reducing germination rates (5–7.5%) compared to zero tillage (17.5–20.0%) [56]. It seems that minimum tillage as well as superficial sowing allow the exposure of seeds to light, which is important for the induction of germination. Montoya-Garcia et al. [39] also recorded similar germination rates under field conditions, between 12.5 and 28.4%. These differences in the literature reports could be due to differences in soil structure or compaction, since the seeds are very small and the energy available for germination may not be enough to sustain seedling emergence [26,56].

Temperatures are also important for plant growth and development. Purslane is an annual summer plant which thrives under high temperatures and dry air conditions. Thermal stress and humid conditions (35 °C and 90% of humidity) may induce the protective mechanisms of the species through the increased production of proline and heat shock proteins, which significantly reduce oxidative damage and preserve photosynthetic activity [61]. On the other hand, the species is sensitive to low temperatures, which define its growing period within the spring to autumn period since, under low temperatures, seed germination is not possible or plants are severely damaged [26]. However, according to Saffaryazdi et al. [62], mild cold stress could be used as a cost-effective tool to increase total phenols, flavonoids and omega-3 fatty acid content through the induction of the biosynthesis of protective secondary metabolites.

### 3.2. Irrigation

Water availability is one of the main factors that affect crop production and distribution of the species in many areas throughout the world. Its deficiency is a primary environmental stress, and the current global climate change situation may cause more frequent, more intense and longer drought periods in already affected regions, such as the Mediterranean basin, while it is expected to expand in other regions of the world [63,64,65,66]. Moreover, scarcity of irrigation water is associated with other environmental stressors, such as soil salinization and increased temperatures, which altogether pose combined stresses and aggravate the threats to crop production and food security through [25,64].

Purslane water requirements are considered low since it is a succulent and drought-tolerant plant. Although it is primary classified as a C4 plant, it is able to shift to CAM-like photosynthesis when subjected to stress and return to C4 metabolism when stress is relieved and plants are rehydrated [67,68,69]. This particular mechanism allows the species to increase its water use efficiency and adapt to drought conditions through the reduction of evapotranspiration during the day and the induction of nocturnal CO_2_ uptake [24].

The changes in the photosynthetic pathways are not the only mechanisms that allow purslane to endure drought stress. D’Andrea et al. [67] suggested that purslane is able to produce osmolytes such as urea or carbohydrates that help to maintain cells’ osmolarity, while several stress-related genes are overexpressed [70]. Similarly, Rahdari and Hoseini [71] and Rahdari et al. [72] reported that the induction of drought stress conditions by the addition of polyethylene glycol up to a concentration of −1 MPa in seeds of purslane resulted in an increase of proline, free sugars and sodium in the emerged plants, although no differences in the germination rates were recorded. This finding indicates the capacity of purslane to maintain turgor pressure under drought conditions even in early plant stages.

The photosynthetic machinery is also protected from oxidative processes caused by ROS production due to water deficit through the induction of biosynthesis of secondary metabolites such as flavonoids and betalains [67,73,74]. However, despite the efficient mechanisms of purslane to endure drought stress, a clear impact on plant development and growth is expected depending on stress duration and severity, as well as the growth stage of plants. Saheri et al. [21] compared three levels of irrigation, e.g., 90% of field capacity (Control), 60% of field capacity and 30% of field capacity after 30 days of germination for 15 days. According to that study, stressed plants showed lower growth rates than the control, despite the activation of resistance mechanisms as recorded by the higher production of flavonoids, phenolic compounds, catalase, proline and soluble sugars [21]. The same authors also recorded a reduction in photosynthesis parameters such as photosynthetic pigment, stomatal conductance and transpiration rate, although no measurements during the night were performed that could show the shift to a CAM-like metabolism. In contrast, Jin et al. [25], who studied purslane tolerance under a continuous drought situation, observed that purslane leaves were able to maintain 90% of their leaf water content (compared to 96% in control plants) after 22 days of water stress, while they quickly recovered to normal levels after one day of rehydration.

Apart from drought, purslane is also able to tolerate excessive irrigation. In particular, Uddin et al. [75] compared different water regimes (e.g., continuous field capacity, continuous saturated conditions, continuous flooded conditions, flooded conditions followed by saturated condition and saturated conditions followed by field capacity) and found that optimal yield was obtained in saturated conditions, whereas the better nutritional values in terms of crude protein and flavonoid content was recorded under flooded conditions.

Ali et al. [51] suggested the use of municipality wastewater for purslane plant irrigation, although special consideration should be given to untreated wastewater in order to avoid public health issues due to contamination. Another approach is the natural irrigation of halophytes with the use of estuarine water in abandoned land areas (e.g., salt pans) in the context of biosaline agriculture [76].

Table 1 compiles the most relevant scientific publications related to the effect of irrigation regime on purslane growth and yield.

The lack of irrigation water is not the only problem that modern agriculture has to tackle. Another important issue is the degradation of the quality of the available water due to soil and aquifer salinization, which severely affects the yield and productivity of several crops and threatens global food security, especially in coastal areas [22,64,78]. Purslane is considered a salt-tolerant species being able to tolerate moderate to high salinity stress, as already confirmed by several studies [79,80]. The germination rates are not severely affected under high salinity levels of 15 dS m^−^^1^ (e.g., 50%), whereas at more severe salinity stress (20 dS m^−^^1^), the germination ratio decreased to 13% [81], while Chauhan and Johnson [56] suggested a decrease of germination ratio by 50% when seeds were germinated under osmotic stress of −0.34 MPa (106 mM of NaCl). Moreover, according to Teixeira and Carvalho [79], who tested the effect of different salt concentrations (0.8 (control), 6.8, 12.8, 24.2 dS m^−^^1^) on purslane growth, suggested that yield was significantly reduced over the control plants only at salinity levels higher than 6.8 dS m^−^^1^, while at the highest salt level a reduction of 29% to 44% was recorded in spring and summer experiments, respectively. Moreover, the same authors suggested that despite the decrease in biomass yield, the level of 6.8 dS m^−^^1^ resulted in higher total lipid content [79]. Similar results were obtained by Alam et al. [82], who treated twelve accessions of purslane with different salinity levels (0, 8, 16, 24 and 32 dS m^−^^1^), including common purslane, and they stated purslane to be a high salinity-tolerant crop. Apart from salinity level, the exposure to salinity conditions is also important for plant response. According to Anastaćio and Carvalho [83], a reduction in yield was observed at 70 mM NaCl or higher, especially when the exposure to salt stress was extended for 20 days or more. Nevertheless, the nutritional properties did not seem to decrease with moderate saline stress, since the accumulation of omega-3 and omega-6 decreased only in higher doses of salinity. Similarly, Yazici et al. [84], who studied the effect of two different saline concentrations (70 and 140 mM NaCl) after exposure for 18 and 30 days, demonstrated that purslane growth was not affected by salinity stress after 18 days, but at higher doses, the fresh weight and relative water content were significantly reduced. Moreover, both treatments induced the antioxidant mechanisms of the species, thus enhancing plants’ antioxidants and proline content. Another possible positive effect of salinity is related to reduced nitrate content, since according to Giménez et al. [85], the combined application of 80 mM NaCl and red–blue and red–blue–far red LED lights improved the yield of purslane microgreens and also reduced the nitrate content of the edible parts. In contrast, they also observed a decrease in flavonoid, chlorophyll and carotenoid compounds compared to the non-saline treatment, which affects the overall nutritional value of the final product.

Table 2 presents the most relevant scientific publications related to the effects of salinity stress on purslane growth and yield.

### 3.3. Mineral Fertilization

Another cultivation practice that is pivotal for the commercial valorization of underutilized/underexplored species is the application of fertilizers. Mineral fertilization was found to be the most frequent fertilization method used in the literature reports, and it has been applied to evaluate not only the growth and yield of purslane but also its nutritional and pharmaceutical quality.

Nitrogen seems to be the most influential nutrient for purslane growth, and different doses of nitrogen have been studied under field conditions. For example, Kaymak [37] applied 150 kg ha^−^^1^ of ammonium nitrate in field-grown purslane plants and obtained a fresh yield of 15.24 ton ha^−^^1^. These results seem to be low since, according to Petropoulos et al. [38] or Karkanis and Petropoulos [77], higher fresh yields can be obtained under no fertilization regime (e.g., 24 and 25 ton ha^−^^1^) in field conditions with two different densities (33 and 240 plants m^−^^2^), a finding which can be explained by the longer growing period in these studies (harvesting at 65 days vs. 35 days). Moreover, genotypic differences could be related to the observed yield differences in the literature reports since, according to Karkanis and Petropoulos [77], a great variation (approximately 15–30 t ha^−^^1^) is expected among purslane germplasm. Similarly, both El-Sherbeny et al. [88] and Montoya-García et al. [39] obtained higher yields with lower nitrogen inputs using ammonium sulfate (84 and 100 kg ha^−^^1^, respectively) and obtained different fresh yields of 11.2–17.6 ton ha^−^^1^ and 60–132 ton ha^−^^1^, respectively. These differences might be again attributed to the difference in harvesting time since the results obtained from the study by El-Sherbeny et al. [88] refer to plants that grew for almost double the time compared to the study of Montoya-García et al. [39], as well as to differences in plant density. The positive correlation of relation biomass production with harvesting time is in accordance with the results obtained by Mortley et al. [89], who harvested plants at 42 d, 63 d and 84 d after sowing and reported values of fresh weights per plant of 1.5 g 7.2, and 27.2 g, respectively. Moreover, Montoya-García et al. [39] reported that apart from nitrogen doses, the number of harvests and plant density may also affect the obtained fresh yields, and further suggested that the optimum combination is the application of 65 kg of N ha^−^^1^, the plant density of 2.500 plants m^−^^2^ and the implementation of three consecutive harvests.

Apart from the positive effects of nitrogen application on yield, special attention should be given to nitrate accumulation in the edible part of the species. According to El-Sherbeny et al. [88] and Montoya-García et al. [39], nitrogen application increases the concentration of nitrate in purslane without exceeding the allowed amounts for fresh consumption. In addition, nitrogen affects purslane’s nutritional properties since it decreases palmitic acid and increases omega-3 fatty acid content while it reduces oxalic acid content, thus improving the overall quality of edible parts [40,90]. Moreover, the same authors suggested that although the application of P and K may not have a significant effect on biomass yield [39], it may regulate the biosynthesis of bioactive compounds such as flavonoids, ascorbic acid or β-carotene and improve the nutraceutical properties of the edible product [90]. According to Kyriacou et al. [91], nitrate content in leafy salads such as purslane can be regulated not only by applying the optimum dose and form of nitrogen but also by the application mode (e.g., base or side-dressing), which affects nitrogen availability throughout the growing season. Finally, Kaşkar et al. [92] suggested significant differences in nitrate accumulation between different genotypes of purslane, a finding which indicates the involvement of nitrate ions in stress protective mechanisms, since nitrate can serve as osmolytes and alleviate negative effects of osmotic stress.

Although nitrogen rates are essential, the nitrogen source is equally important for purslane growth and yield, as well as for the quality of the final product. For example, Kaymak [37] compared different nitrogen sources under field conditions (e.g., urea, calcium ammonium nitrate, ammonium sulphate and ammonium nitrate) using the same N dose and reported that urea and ammonium nitrate were the most efficient nitrogen sources for improved purslane yield (13.85 and 15.24 tons ha^−^^1^, respectively) without resulting in high nitrate accumulation in leaves. Moreover, Fontana et al. [40] suggested that the ratio of nitrate and ammonium may affect yield and growth of purslane, with the ratio of 40:60 for NO_3_^−^ N/NH_4_^+^ being the optimum one for obtaining the highest yield and quality. In contrast, Szalai et al. [93] reported that the increase of ammonia application even at a ratio of 75:25 for NO_3_^−^ N/NH_4_^+^ may decrease yield and plant quality; however, in order to avoid negative effects of a high ammonium rate, the same authors recommended harvesting purslane plants at the stage of 10 to 15 true leaves when the inhibitory effects of ammonium are not yet a limiting factor. Similar results were obtained by Camalle et al. [94], who tested three different NO_3_^−^ N/NH_4_^+^ ratios (e.g., 100:0, 66:33, 25:75 of NO_3_^−^ N/NH_4_^+^) and two levels of salinity (0 and 50 mM NaCl), and although they recorded higher yields with higher NO_3_^−^ levels (e.g., 100:0 and 66:33 of NO_3_^−^ N/NH_4_^+^), they also observed severe leaf chlorosis and higher oxalic acid content for the same treatments. Moreover, the same authors suggested that increasing salinity (50 mM of NaCl) alleviated the deleterious effects of NH_4_^+^ and also improved the nutritional properties of the edible leaves through the increase of crude proteins, total fatty acids, carotenoids and total chlorophyll content [94]. Therefore, despite the lower yield, the application of high amounts of NH_4_^+^ nitrogen (33% to 75% of total nitrogen) along with moderate salinity stress could be recommended for the optimum quality without severely compromising purslane yield.

### 3.4. Planting and Harvesting Time

Sowing date and harvesting are two agronomic practices that may have an impact on yield and quality of the final product. Considering the short growth cycle of the species, sowing can take place on various dates from early May to mid-June, as soon as the temperature requirements for germination are fulfilled, and even more than one cropping is possible within the same growing period [28,38,88], while plant density may also affect biomass yield [95,96]. The sowing of seeds with the existing sowing machines is troublesome due to the small size of seeds, while seed pelleting would significantly increase seed and crop production cost. Therefore, Brennan [97] developed a slide hammer seeder, which is a novel machine that allows uniform dispersing of small-seeded species without increasing production cost. Regarding the harvesting of the species, single or multiple harvesting can be performed, depending on the market requirements and the desired composition of the edible portions of the species. Higher yields are expected with longer growing periods, although a decrease of the nutritional properties of purslane is recorded [39,93]. According to Petropoulos et al. [28], harvesting at 29, 43 or 52 days after sowing (DAS) resulted in significant differences in the chemical composition of leaves and stems. In particular, omega-3 fatty acid content was higher at early to mid–late harvesting (e.g., 29 and 42 DAS), whereas oxalic acid and oleracein content increased at late harvesting. Similar results were reported by Mortley et al. [89], who also detected higher amounts of fatty acids (mainly α-linolenic, linoleic acid and palmitic acid) at 60 days after transplantation (DAT), compared to earlier harvesting at 20 and 40 DAT. Plant phenological stages could be another criterion for purslane harvesting, since according to Saffaryazdi et al. [98] significant differences in total phenols and the main fatty acid (α-linolenic and linoleic acid) content in leaves and stems was recorded between the vegetative and the flowering stage. In particular, an increase of total phenols and fatty acids was recorded in leaves at the flowering stage, whereas the opposite trend was suggested for total phenol content in stems (fatty acid content was not affected). In contrast, Uddin et al. [99] recorded an increase of total phenols content at late harvesting stages which was also associated with higher antioxidant activity determined with FRAP and AEAC assays.

Table 3 and Table 4 compile the most relevant scientific research reports related to chemical fertilization, including information regarding the nutrient form and dose applied, the cropping system (pots, hydroponic or soilless), the obtained yield, the harvesting time and the plant density.

## 4. Sustainable Practices and Cropping Systems

Overall, few studies have tested the effect of sustainable agricultural practices in purslane yield and chemical composition or their influence on soil recovery. The most common sustainable practice studied in purslane cultivation in the current bibliography is the application of animal manure. In particular, Fallah and Omrani [101] evaluated the substitution of mineral fertilizers with similar rates of organic ones (e.g., N- and P-based broiler litter and cattle manure at rates of 120 kg ha^−^^1^ and 50 kg ha^−^^1^ for nitrogen and phosphate, respectively). The authors of the study reported that although mineral fertilizer treatments provided higher amounts of nitrogen in a shorter time period, they also led to accumulation in the form of nitrate in leaves and to reduced nitrogen absorption in the structure of plant organic compounds, indicating that a balanced nutrient supplementation is needed to reach both high yield and quality in purslane. They also concluded that an organic nitrogen source is a more effective and sustainable strategy for purslane production. In the study of Hosseinzadeh et al. [35], the combined effect of water stress, arbuscular mycorrhiza fungi (*Rhizophagus irregularis*) inoculation and fertilization with organic and mineral fertilizers on purslane growth and nutritional value was investigated. The results of the study showed that the substitution of organic fertilizers by 75% with farmyard manure significantly increased AMF colonization in both water stress and normal irrigation conditions, while the substitution of organic fertilizers with farmyard manure by 50–75% also increased grain and biomass yield and α-linolenic acid content in seeds and leaves [35,102]. Moreover, the application of farmyard manure contributed to the improvement to soil quality, as indicated by pH reduction and the increase of organic carbon content and the cation exchange capacity, while it alleviated the negative effects of water stress.

Yang et al. [103] studied how different doses of an organic mineral fertilizer (OMF) based on chicken manure, K-feldspar powder and different microbial agents may improve the soil fertility in soils over-supplied with chemical fertilizers by growing purslane as a test plant for 80 days. According to the results of the study, the plants grown in over-supplied soils with no OMF addition were not able to survive even 15 days after transplantation due to a rapid decrease of pH and an increase in electrical conductivity. On the other hand, plants grown in over-supplied soils amended with gradients of OMF showed a higher or similar to untreated plant biomass yield and improved nutritional quality, while plants treated only with OMF showed a significantly higher yield than the rest of the treatments, especially at high application rates [103]. Moreover, OMF significantly improved soil quality parameters such as enzyme activities and the C/N ratio, thus allowing purslane plants to recover and overcome the unfavorable conditions due to excessive fertilization rates.

Other practices include the biofortification of plants through tailor-made nutrient solutions. For example, D’Imperio et al. [104] suggested the boron biofortification of purslane through a soilless cultivation system, a practice that could contribute to the fulfillment of daily boron requirements by consuming 48 g to 75 g of fresh purslane. Moreover, D’Imperio et al. [105] reported the benefits from a foliar application of Zn, which increased not only Zn content in the edible parts of the plant but also increased phytochemical content such as carotenoids and fatty acids. Finally, Puccinelli et al. [106] proposed the Se biofortification of purslane microgreens through the enrichment of a nutrient solution with Na_2_SeO_4_ at various rates.

The phytoremediation of degraded soils with purslane cultivation has also shown promising results due to the tolerance of the species to stressful conditions, including those of excessive contaminants. Several studies highlighted the remediative properties of the species from excessive heavy metal content such as Cr [107,108,109], Ni and Cd [52] or others [110,111,112]. Moreover, Abdallah et al. [113] suggested the removal of macro-nutrients from sewage sludge dump sites in order to mitigate eutrophication in sludge-amended soils. Yadav et al. [114] also reported the use of purslane for the ecological restoration of fly ash disposal areas, which constitute one of the most hazardous dumping sites.

Regarding the cropping systems suggested for purslane cultivation in the literature, several options are available. Most of the studies refer to soil growing medium, either directly in the field [77] or in pots under protected environment conditions [98,109]. Experiments in the field showed that purslane is a promising candidate for exploiting degraded soils and/or irrigation water of low quality. Moreover, it can be integrated into intercropping schemes that can improve soil fertility and physicochemical qualities [95,115] or used as a cover crop (mulching) that improves soil enzyme activity [116]. On the other hand, the advantage of pot cultivation is related to the reduced growth cycle and the control of environmental conditions which allow several cropping cycles over the year. Apart from pot cultivation, hydroponic systems in the form of floating culture [92,104,117] or other soilless systems [118,119] have also been suggested. Such systems offer the option of increased plant densities for the production of microgreens [85,105,106] or sprouts [120].

## 5. Agronomic Recommendations

Depending on the chosen cropping system, the edaphoclimatic conditions and inputs applied by the farmers, purslane yield may vary greatly. The interest of farmers for purslane cultivation could be driven by its high nutritional value, its high tolerance to stressors like salinity or drought and its low fertilization requirements to obtain high yields. In terms of stress tolerance, purslane has been classified as a halophyte, being able to thrive in high salinity conditions as 50 mM NaCl or 5 dS/m^−^^1^ and even to be moderately productive at higher salinity levels of 150 mM NaCl or 15 dS/m^−^^1^, while improving its nutritional properties. This property is of great importance for farmers whose soils or irrigation water are affected by salinization, thus being an alternative for them to commercially exploit low quality soils.

Low inputs are also suggested to obtain high yields in purslane cultivation. In particular, irrigation needs are low since purslane is a succulent C4–CAM plant adapted to arid conditions, able to grow under severe drought stress. It can complete its growth cycle without additional irrigation during the summer in hot climates, such as those prevailing in the Mediterranean basin. Nevertheless, irrigation would enhance purslane’s yield in such conditions, giving farmers the opportunity to improve expected yields with low water usage and also increase the water use efficiency of the crop.

Another important perspective is the nutrient requirements of purslane in order to maximize yield. Purslane needs low fertilizer inputs in general, but it mostly responds to the application of nitrogen. In particular, it seems to respond better to nitrogen when applied in nitrate and ammonium form rather than other forms such as urea. Additionally, the proportion of nitrate and ammonia and the optimal total nitrogen rates for high yields are not clearly defined, since there are studies proposing low nitrate:ammonium nitrogen proportions (40:60) for higher productions [40], whereas other reports suggest that higher nitrate rates are beneficial to yield (75:25 to 66:33) [93,94]. Nevertheless, ammonia use is generally recommended under salinity conditions to improve stress tolerance and production [94]. Some studies have obtained good fresh yield results with low or no nitrogen applied under field conditions (6 to 24 ton ha^−^^1^) [37,38]; although, with relatively low nitrogen inputs such as 60 kg N ha^−^^1^, purslane fresh yield could increase up to 120 ton ha^−^^1^ [36,39,88].

Harvesting time and the density of the plantation are also important for field production, while they may affect the nutritional quality of purslane. Low densities produce bigger plants [88], whereas higher densities result in smaller and lower total yield [39]; however, when similar fertilization and harvesting time were considered, both experiments produced similar fresh yields of around 120 ton ha^−^^1^. Similarly, Karkanis and Petropoulos [77] and Petropoulos et al. [38] also obtained similar fresh yields of 25 and 24 ton ha^−^^1^, respectively, with different sowing densities (240 plants m^−^^2^ and 33 plants m^−^^2^, respectively), although the growing period was different (e.g., 45 days and 65 days after sowing, respectively), suggesting that higher densities might be more productive in shorter periods of time. Thus, the harvesting time may have a great impact on yield. The growing periods studied were around 20 to 60 days after sowing, and generally higher yields were obtained with longer periods; however, the nutritional and product quality must be also considered. It seems that longer growing periods (harvesting at 40 and 60 days after sowing) also produce higher amounts of omega-6 and omega-3 fatty acids but harvesting at 20 days after resulted in a better ratio of omega-6 and omega-3 fatty acids [89]. In addition, the ability of purslane plant to regrow after harvest allows harvesting multiple times, thus increasing total yield [41].

## 6. Future Prospects and Conclusionary Remarks

**Figure 1:**
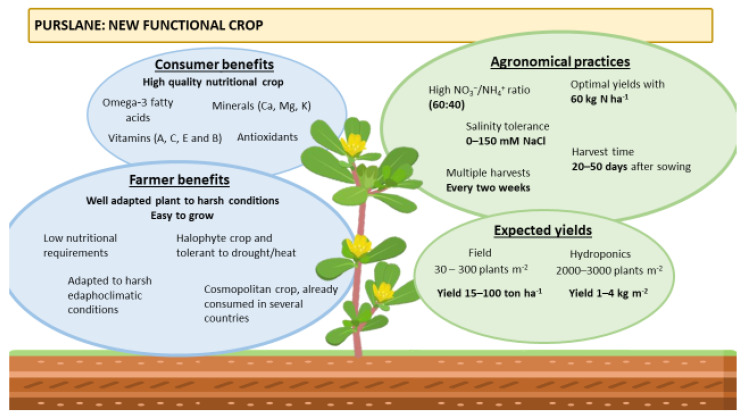
The concept of introducing purslane as a new functional crop.

The degradations of soils, the scarcity of water and increasing temperatures are the main problems that affect conventional agriculture. The introduction of new/alternative species in the existing farming systems are pivotal for the sustainability of agroecosystems and the development in rural areas. Apart from the primary sector, the food industry may also benefit from the valorization of such species through the development of functional and healthy foods, while the adoption of a circular economy approach may provide new antioxidant compounds from the byproducts of crops and the processing sector. Figure 1 presents the concept of introducing purslane as a new functional crop.

Recently, Di Cagno et al. [121] introduced a novel fermented beverage from purslane juice with an enhanced content of vitamins and phenolic compounds and ameliorating effects against intestinal inflammation and epithelial injury. Apostol et al. [122] reported that the incorporation of purslane leaves in tomato sauce significantly improved the quality and omega-3 fatty acid content without compromising other quality features or the rheological properties of the final product. The addition of microencapsulated purslane seed oil in mango juice improved the viscosity of the final product and its nutritional value through the increase of omega-3 fatty acid content. The use of purslane extract in edible packaging films also showed promising results against the microbiological and oxidative damage of sausages, while similar effects were reported by Pratiwi et al. [123] for the packaging of mozzarella cheese. Moreover, Rayan et al. [96] suggested the development of novel proteins from purslane seed flour and its incorporation in food proteins, while Delvarianzadeh et al. [124] reported the use of purslane powder in the preparation of voluminous bread. The use of purslane powder obtained from stems and stalks was also evaluated for the biofortification of durum wheat pasta and wheat bread with omega-3 fatty acids by Melilli et al. [125,126], respectively; while Santiago-Saenz et al. [127] suggested the use of mixtures of dried edible parts of various wild edible plants, including purslane, in the preparation of food supplements and food products.

Several new compounds with bioactive potential have been isolated from purslane during recent years, indicating the high antioxidant potential of the species. For example, new alkaloids with anti-inflammatory activity were identified in various studies [128,129,130,131,132], while Wang et al. [133] detected two new lignans with similar properties. These novel compounds could be isolated and incorporated in the design of new drug formulations, nutraceutical and pharmaceutical products, thus increasing the added value of purslane. Moreover, Hoseini et al. [134] suggested the valorization of purslane seeds for biodiesel production and as feedstock, while Petropoulos et al. [135] reported the high potential of using seeds, seed oils and processing by-products (seedcakes) as novel sources of omega-3 fatty acids in the food industry. All these aspects highlight the multifaceted prospects of purslane, which may result in high added value products and ensure the income of farmers, especially in the small-scale farms of the Mediterranean basin.

In conclusion, the need of using new alternatives species able to adapt under climate-changing conditions in order to replace conventional plants in those areas where cropping is not profitable in the short- and long-term is a major priority. Wild edible plants are potential candidates as new crops, and purslane has been proposed as a “future crop” due to its high nutritional value, omega-3 fatty acid, bioactive compound and antioxidant content and its high adaptability to different stressors such as heat, drought, salinity or soil degradation. Therefore, the presented information would be useful for farmers and could be used as best practice guides in order to achieve the highest yields and the best quality of the final product. Considering the wild nature of the species, the development of best practice guides is needed to update the management, yield, nutritional properties and tolerance to stress; new sustainable practices in purslane cultivation such as compost application, bio stimulants or bio fertilizers should be further evaluated in order to determine their effect on biomass yield and/or the nutritional quality of purslane, aiming at land reclamation and degraded soil recovering.

## Figures and Tables

**Table 1:** *Portulaca oleracea* L. fresh yield under different water conditions.

Cultivation Conditions	Water Stress	Duration (Days)	Plant Density	Fresh Yield		Country	References
Field experiment (June to July, 2016); sandy clay loam soil, with pH 7.4 and 1.3% of organic matter; mean daily temperatures of 28 °C; precipitations of 12 mm during the experiment; plants harvested at 45 DAS.	Mediterranean arid conditions	45	240 plants m^−2^	25 ton ha^−1^	10.42 g plant^−1^	Greece	[77]
Pots experiment (July to August, 2015); plants grown in 20 cm × 30 cm (d/h) pots with soil pH 5.23; EC 1.15 dS m^−1^ and organic matter content of 22.02%; three plants per pot; plants harvested at 30 DAE.	Field capacity	30 no stress	-	3.01 kg m^−2^ **	91.26 g plant^−1^	Malaysia	[75]
	Continuous saturated	30 stressed	-	2.28 kg m^−2^ **	68.97 g plant^−1^		
	Continuous flooded	30 stressed	-	1.41 kg m^−2^ **	42.67 g plant^−1^		
	10 days flooded and saturated next	10 flooded/20 saturated	-	1.57 kg m^−2^ **	47.67 g plant^−1^		
	10 days saturated and field capacity next	10 saturated/no stress	-	2.74 kg m^−2^ **	82.93 g plant^−1^		
Greenhouse experiment; plants grown in 10 cm × 15 cm (d/h) pots with hummus, clay and sand (2:1:3); average day/night temperatures (25/18 °C); relative humidity (60/70%) and photoperiod (16/8 h); one plant per pot. Plants harvested at 45 DAE.	Control (90% field capacity)	45 no stress	-	0.45 kg m^−2^ **	13.70 g plant^−1^	Iran	[21]
	Mild drought (60% field capacity)	30 no stress/15 stressed	-	0.32 kg m^−2^ **	9.83 g plant^−1^		
	Severe drought (30% field capacity)	30 no stress/15 stressed	-	0.24 kg m^−2^ **	7.23 g plant^−1^		

DAS (days after sowing), DAE (days after emergence). For yield comparison, we assumed a density of 33 plants m^−2^ (yields estimated according to this assumption are indicated with double asterisk (**).

**Table 2:** *Portulaca oleracea* L. fresh yield under different salinity stress levels.

Cultivation Conditions	Salinity Stress	Duration (Days)	Plant Density	Fresh Yield		Country	References
Glasshouse experiment (July to October 2013); 10-day-old seedlings were transplanted to 10 L pots with rice field soil; pH 4.8; 2.64% organic carbon; 1.25 g cc1bulk density and CEC of 7.06 me 100 g^−1^ soil; one plant per pot; saline treatment applied with NaCl; plants harvested at 60 DAT.	0 dS m^−1^	60 no stress	-	42.75 ton ha^−1^ **	129.48 g plant^−1^	Malaysia	[82]
	8 dS m^−1^	29 no stress/30 stressed	-	37.29 ton ha^−1^ **	112.94 g plant^−1^		
	16 dS m^−1^	29 no stress/30 stressed	-	34.71 ton ha^−1^ **	105.14 g plant^−1^		
	24 dS m^−1^	29 no stress/30 stressed	-	30.83 ton ha^−1^ **	93.40 g plant^−1^		
Greenhouse experiment (January to March, 2016); plants grown in 3 L pots with 1 kg of soil; pH 5; average day/night temperatures (22.2/17.9 °C); relative humidity from 12.9% to 88.3%; one plant per pot; saline treatment applied with NaCl; plants harvested at 50 DAS.	0	50 no stress	-	16.5 ton ha^−1^ **	50 g plant^−1^	France	[80]
	5 dS m^−1^	4 no stress/46 stressed	-	14.85 ton ha^−1^ **	45 g plant^−1^		
	9.8 dS m^−1^	4 no stress/46 stressed	-	12.54 ton ha^−1^ **	38 g plant^−1^		
	20 dS m^−1^	4 no stress/46 stressed	-	5.94 ton ha^−1^ **	18 g plant^−1^		
Greenhouse experiment (April to May 2017); plants grown in 26 mL capacity cells in floating system with nutritive solution; pH 7.7; day/night temperatures (38.1/13.6 °C) and relative humidity from 10% to 85%. Saline treatment applied with NaCl; plants harvested at 50 DAS.	2.5 dS m^−1^	22 no stress/28 stressed	3500 plants m^−2^	3.84 kg m^−2^	1.097 g plant^−1^ **	Spain	[81]
	5 dS m^−1^	22 no stress/28 stressed	3500 plants m^−2^	3.58 kg m^−2^	1.02 g plant^−1^ **		
	7.5 dS m^−1^	22 no stress/28 stressed	3500 plants m^−2^	3.25 kg m^−2^	0.927 g plant^−1^ **		
	10 dS m^−1^	22 no stress/28 stressed	3500 plants m^−2^	3.24 kg m^−2^	0.926 g plant^−1^ **		
	15 dS m^−1^	22 no stress/28 stressed	3500 plants m^−2^	2.88 kg m^−2^	0.822 g plant^−1^ **		
Greenhouse experiment (2018); plants grown in artificial soil with Hoagland´s solution; day/night temperatures (28.6/19.8 °C) and relative humidity (76.8%/82.4%); one plant per pot; saline treatment applied with NaCl when plant height reached 15 cm; harvest took place 14 days after.	0 mM	14 no stress	-	0.43 kg m^−2^ **	13.3 g plant^−1^	China	[86]
	50 mM	14 stress	-	0.46 kg m^−2^ **	14.0 g plant^−1^		
	100 mM	14 stress	-	0.42 kg m^−2^ **	12.6 g plant^−1^		
	150 mM	14 stress	-	0.29 kg m^−2^ **	8.7 g plant^−1^		
	200 mM	14 stress	-	0.25 kg m^−2^ **	7.6 g plant^−1^		
Greenhouse experiment (2020); plants grown in 0.5 L pots with peat:vermiculite:perlite (50:25:25) and Hoagland´s nutrient solution; day/night temperatures (23/17 °C); photoperiod (16/8 h) and relative humidity ranged between 50 and 80%; one plant per pot; saline treatment applied with NaCl; plants harvested at 77 DAS.	0 mM	77 days	-	2.064 kg m^−2^ **	62.55 g plant^−1^	Spain	[87]
	100 mM	42 no stress/35 stressed	-	1.86 kg m^−2^ **	56.3 g plant^−1^		
	200 mM	42 no stress/35 stressed	-	1.28 kg m^−2^ **	38.78 g plant^−1^		
	400 mM	42 no stress/35 stressed	-	0.62 kg m^−2^ **	18.77 g plant^−1^		

DAS (days after sowing), DAT (days after transplanting). For yield comparison, we assumed a density of 33 plants m^−^^2^ (yields estimated according to this assumption are indicated with double asterisk (**).

**Table 3:** The effect of different nitrogen forms, rates and density of plants in yield experiments with *Portulaca oleracea* L.

Cultivation Conditions	Nitrogen Form	Nitrogen Dose	Plant Density	Fresh Yield		Country	References
Field experiment (June to July, 2016); sandy clay loam soil, with pH 7.4 and 1.3% of organic matter; mean daily temperatures of 28 °C; precipitations of 12 mm during the experiment; plants harvested at 45 DAS.	None	None	240 plants m^−2^	25 ton ha^−1^	10.42 g plant^−1^	Greece	[77]
Field experiment (May to July, 2014); loam soil with pH 7.4 and 1.3% organic matter content; mean daily temperatures of 25 °C; plants harvested at 65 DAS.	None	None	33 plants m^−2^	24 ton ha^−1^	72 g plant^−1^	Greece	[38]
Field experiment (June to July, 2009 and 2010); sandy loam soil, pH 7.23, organic matter content of 1.16% and total nitrogen of 0.06%; plants harvested at 35 DAS; the obtained yield was the mean of both years.	None	None	-	5.46 ton ha^−1^	16.54 g plant^−1^ **	Turkey	[37]
	NH_4_NO_3_	150 kg N ha^−1^	-	12.71 ton ha^−1^	38.51 g plant^−1^ **		
	Urea	150 kg N ha^−1^	-	11.54 ton ha^−1^	34.96 g plant^−1^ **		
	(NH_4_)_2_SO_4_	150 kg N ha^−1^	-	10.8 ton ha^−1^	32.72 g plant^−1^ **		
	(Ca(NH_4_NO_3_)_2_)	150 kg N ha^−1^	-	10.92 ton ha^−1^	33.09 g plant^−1^ **		
Field experiment (June 2007 and 2008); clay soil with average pH 7.70, electical conductivity of 2.5 dS m^−1^, 0.50% organic matter content and 0.05% of total N; plants harvested at 61 DAS.	(NH_4_)_2_SO_4_	49 kg N ha^−1^	16 plants m^−2^	115.40 ton ha^−1^	721.30 g plant^−1^	Egypt	[88]
	(NH_4_)_2_SO_4_	73 kg N ha^−1^	16 plants m^−2^	140.90 ton ha^−1^	880.80 g plant^−1^		
	(NH_4_)_2_SO_4_	98 kg N ha^−1^	16 plants m^−2^	159.77 ton ha^−1^	998.60 g plant^−1^		
Field experiment (July to August, 2014); sandy clay soil with pH 8.2; maximum day tempertures ranged from 19 °C to 30 °C and minimum temperatures ranged from 6 °C to 11 °C; plants harvested at 42 DAS.	None	None	1750 plants m^−2^	110 ton ha^−1^	6.28 g plant^−1^	Mexico	[39]
	(NH_4_)_2_SO_4_	100 kg kg N ha^−1^	1750 plants m^−2^	120 ton ha^−1^	6.86 g plant^−1^		
	NH_4_H_2_PO_4_	200 kg N ha^−1^	1750 plants m^−2^	126 ton ha^−1^	7.20 g plant^−1^		

DAS (days after sowing), DAE (days after emergence), TLS (true leaf stage). For yield comparison, we assumed a density of 33 plants m^−2^ (yields estimated according to this assumption are indicated with double asterisk (**).

**Table 4:** The effect of different nitrogen forms and rates in hydroponics and pot experiments with *Portulaca oleracea* L.

Cultivation Conditions	Nitrogen Form	Nitrogen Dose	Plant Density	Fresh Yield		Country	References
Hydroponic experiment conducted in greenhouse conditions (July 2004) in trays with peat floating on the nutrient solution; day/night temperatures (35/15 °C); plants harvested at 20 DAS.	None	None	3105 plants m^−2^	0.27 kg m^−2^	0.09 g plant^−1^	Italy	[40]
	NO_3_^−^/NH_4_^+^ (40:60)	12 mmol L^−1^	3105 plants m^−2^	1.39 kg m^−2^	0.45 g plant^−1^		
	NO_3_^−^/NH_4_^+^ (40:60)	24 mmol L^−1^	3105 plants m^−2^	1.50 kg m^−2^	0.48 g plant^−1^		
	NO_3_^−^/NH_4_^+^ (40:60)	36 mmol L^−1^	3105 plants m^−2^	1.81 kg m^−2^	0.58 g plant^−1^		
Hydroponic experiment conducted in greenhouse conditions (July 2004) in trays with peat floating on the nutrient solution; day/night temperatures (35/15 °C); plants harvested at 20 DAS.	NO_3_^−^/NH_4_^+^ (60:40)	12 mmol L^−1^	3105 plants m^−2^	1.38 kg m^−2^	0.40 g plant^−1^		
	NO_3_^−^/NH_4_^+^ (40:60)	12 mmol L^−1^	3105 plants m^−2^	1.48 kg m^−2^	0.48 g plant^−1^		
	NO_3_^−^/NH_4_^+^ (0:100)	12 mmol L^−1^	3105 plants m^−2^	0.71 kg m^−2^	0.23 g plant^−1^		
Hydroponic experiment conducted in greenhouse (September 2007) in trays with vermiculite floating on the nutrient solution, pH 7.7, EC 0.85 dS m^−1^; plants were harvested at 13 DAS.	NO_3_^−^/NH_4_^+^ (60:40)	80 mmol L^−1^	3200 plants m^−2^	1.38 kg m^−2^	0.43 g plant^−1^	Spain	[92]
Hydroponic experiment conducted in greenhouse conditions from February to July in trays with tuff:peatmoss (2:1); maximun temperature 28 °C; total yield of plants harvested five times during 60 days.	Clark´s nutrient solution	22 mM NO_3_^−^ and 2.78 mM NH_4_^+^	-	26.92 kg m^−2^	8.97 g plant^−1^ **	Jordan	[41]
Greenhouse experiment; plants cultivated in 8 L styrofoam boxes with organic substrate (forest residue, compost and white peat), pH 6, EC 2 mS cm^−1^, total nitrogen 300 mg L^−1^; day/night temperature (33.7/16.7 °C) and relative humidity (49.7%/12.2%); plants harvested at 31 DAS.	None	None	2200 plants m^−2^	2.38 kg m^−2^	1.08 g plant^−1^	Portugal	[36]
	NH_4_NO_3_	30 kg N ha^−1^	2200 plants m^−2^	4.01 kg m^−2^	1.82 g plant^−1^		
	NH_4_NO_3_	60 kg N ha^−1^	2200 plants m^−2^	5.10 kg m^−2^	2.32 g plant^−1^		
	NH_4_NO_3_	90 kg N ha^−1^	2200 plants m^−2^	5.30 kg m^−2^	2.41 g plant^−1^		
Plants grown on October in 2 L pots with peat and perlite (2:1 *v*/*v*), pH 6, EC 0.35 dS m^−1^, total nitrogen 0.14%; plants harvested at 37 DAS.	NO_3_^−^/NH_4_^+/^Urea	None	-	1.10 kg m^−2^ **	33 g plant^−1^	Greece	[100]
	NO_3_^−^/NH_4_^+/^Urea	200 mg N L^−1^	-	2.50 kg m^−2^ **	75.7 g plant^−1^		
	NO_3_^−^/NH_4_^+/^Urea	400 mg N L^−1^	-	4.19 kg m^−2^ **	126.9 g plant^−1^		
	NO_3_^−^/NH_4_^+/^Urea	600 mg N L^−1^	-	7.24 kg m^−2^ **	219.4 g plant^−1^		

DAS (days after sowing). When the density is unknown, we assume a density of 33 plants m^−2^ for pot experiments and 3000 plants m^−2^ for hydroponic experiments. Yields with assumed densities are signalized with double asterisk (**).

## Data Availability

Not applicable.
